# Chancre in the Urethra

**Published:** 1844-07-13

**Authors:** 


### CHANCRE IN THE URETHRA.

The Provincial Medical Journal contains a clinical lecture by Mr. Parker on chancre in the urethra, illustrated by cases. The first of these alluded to by Mr. Parker, is that of a Scotch hawker, twenty-nine years of age, who contracted, seven years previously, sores on the penis, and a running from the urethra, from the same connexion. The discharge from the urethra became permanent, and constitutional symptoms also showed themselves, and his wife also became affected therewith. Three of his children died a few months after birth, presenting symptoms of secondary syphilis. When the man came under Mr. Parker's care he was in the following state:—the head and face were covered with foul blotches, consisting of incrustations or scabs, concealing deep, irregular, and ill-conditioned ulcers; there was superficial redness of the fauces, but no ulceration, and he was feeble and emaciated from long continued disease. The skin disease was evidently pustular in its commencement, as one or two freshly formed pustules were on the face; these were situated on an inflamed base, and when they broke and discharged, run into ulcers, covered with flat or conical crusts, thus constituting a variety of disease, to which the term "pustulo-crustaceous" has been applied. On examination, a sanious oozing was perceived from the urethra, very different from that which charcterizes chronic gonorrhœa or gleet. About an inch from the meatus, immediately behind the glans penis, existed a circumscribed induration, about the size of a hazel-nut; this was painful when pressed between the fingers, and the pressure occasioned some blood and portions of white tenacious sloughs to issue from the urethra. On separating the lips of the meatus urinarious, by means of a speculum made for the purpose, the commencement of the ulceration, which appeared to extend deeply into the urethra, could be perceived. This case Mr. Parker rightly considers to be interesting, from the great length of time that the chancre existed in the urethra as a primary venereal sore, and from the fact that the external sores, and the secondary symptoms, were subjected to repeated treatment, apparently successfully, while the ulcer in the urethra remained totally unaffected by the remedial measures which were employed. The result of this case is not mentioned by Mr. Parker, who proceeds in lieu thereof to describe the sy[illegible]ptoms presented by chancre in the urethra. These are heat, itching or irritation in the canal, occurring after a suspicious connexion, unaccompanied by discharge; pain or tenderness in a particular part of the urethra when it is rolled between the fingers: the presence of a distinct induration at the point where the pain is complained of; pain also increased during micturition, and referred to the same point. Discharge from the urethra occurs at various and irregular periods after the setting in of the first symptoms; it is very different from the discharge of gonorrhœa; it may be sanious, bloody, or of a sloughing character, and commonly does not flow unless the indurated portion be pressed forcibly between the fingers. The only disease for which chancre in the urethra may be mistaken is gonorrhœa, from which it may be distinguished by the history of the case, character and quantity of the discharge, the presence of a distinct circumscribed induration in some part of the urethra, most commonly seated in or immediately behind the glans penis. This circumscribed induration must not be mistaken for that general induration of the corpus spongiosum urethræ which accompanies acute gonorrhœa, and results from an effusion of lymph, &c., into the cells of the spongy body. This state in gonorrhœa is generally accompanied by chordee, a symptom absent in chancre of the urethra. The prognosis of chancre in the urethra is not always favourable; two cases are published by Ricord, in which a fatal termination took place by extension to the bladder. The prognosis is again unfavourable as regards the integrity of the organs of generation, since, however carefully they may be watched, severe mutilations are occasionally produced. The following are the principal evils produced by chancre in the urethra:—contraction of the orifice by the cicatrix of the chancre, or of the canal itself from the same cause; perforation of the canal, or its destrution to a greater or less extent. The orifice has been so contracted as not to admit the bulbous extremity of an ordinary silver probe. Perforations are variable in extent and situation, but are commonly found immediately behind the glans. A very common seat of chancre is the fossa navicularis, and the glans is sometimes scooped out as it were by the spreading of venereal ulceration in this situation. The destruction of a portion of the canal takes place when the disease assumes a phagedenic form, and spreads by rapid ulceration or sloughing. It is not however so likely to happen, if the disease has been at once diagnosticated and properly treated from the commencement. With respect to the treatment, Mr. Parker lays great stress upon absolute confinement of the patient to bed, and the recumbent position. He says it is the most adjuvant in the treatment of all primary and even constitutional syphilitic diseases. The diet must also be of the most simple kind. With these aids the treatment becomes easy, and is very generally successful. Mild opiates and mercurials, with simple astringent injections, will in almost all cases effect a cure, but it is next to impossible to cure these sores while the patient goes about and pursues his ordinary occupations, even in a minor degree.

